# Risk assessment of chronic poisoning among Indian metallic miners

**DOI:** 10.4103/0019-5278.55121

**Published:** 2009-08

**Authors:** Sarang V. Dhatrak, Subroto S. Nandi

**Affiliations:** Department and Institution: Occupational Medicine, National Institute of Miners' Health, JNARDDC Campus, Wadi, Nagpur - 440023, India

**Keywords:** Environmental health, metallic minerals, miners, chronic poisoning, toxicants

## Abstract

The estimated average daily employment in the Indian mining sector is 5,60,000, which comprises 87% in the public sector and 13% in the private sector, of which around 70,000 are working in metallic mines. The mine workers are exposed to dust of various potentially toxic substances. The common toxicants present in the mining environment are lead, mercury, cadmium, manganese, aluminium, fluoride, arsenic, etc. Inhalation and absorption through the skin are common routes of exposure. Low-dose chronic exposure of toxic substances results in the accumulation of toxicants in the body. Hence, there is a need to monitor the mining environment as well as the miners for these toxicants.

## INTRODUCTION

India is rich in mineral resources which are spread over diverse geographic locations. In India, the mining sector employs nearly 1.5 million people directly/indirectly. India produces 64 minerals: four fuel minerals, ten metallic minerals, and 50 nonmetallic minerals. The Indian mining industry is characterized by a large number of small operational mines; there are approximately 3,100 functioning mines in the country. Six hundred of these mines produce coal and lignite, 500 mines produce metallic minerals, and 2,000 mines produce nonmetallic minerals. Eight hundred of these mines are in the public sector and 2,300 mines in the private sector.[1] India is self-sufficient in about 27 minerals. In world production, India ranked 2^nd^ in barites, chromite, and talc, 3^rd^ in coal and lignite, 4^th^ in iron ore and kyanite, 6^th^ in bauxite, and 7^th^ in manganese in 2002-03. The estimated average daily employment in the mining sector is 5,60,000, 4,90,000 (87%) in the public sector and 70,000 (13%) in the private sector,^2^ of which around 70,000 work in metallic mines.

The extraction of mineral resources from the earth's crust is seldom easy. Mineral production can be divided into two general phases: the extraction phase which is concerned with removing material from the earth, and the processing phase which separates the mineral component from the ore. Commonly, the word, “mining” is used to describe the phase of extraction, whereas processing describes the phase of preparing the product for human use. Usually the people involved in both are called miners or mineworkers. In its simplest definition, mining includes all of the job processes required to produce a mineral commodity in its saleable form.

The ore contains principal mineral plus trace amounts of other potentially toxic material. Ore is processed by crushing, screening, washing etc. Workers are thus exposed to dust of various potentially toxic substances during the processing of ore. The mine worker is exposed to large amount of dust for eight hours/day and six days/week over a lifetime of work. In the mining environment, dermal and inhalational exposures predominate and the conditions of work often intensify exposure due to increased respiration rates and repetitive exposure to toxicants. This results in the accumulation of toxicants in the body over many years. The most commonly studied occupational health problems among miners all over the world are silicosis, coal workers' pneumoconiosis, asbestosis, etc. but there are very few studies which describe the ill effects of toxic materials present in the mining environment. This article addresses the issues of exposure and poisoning among metallic miners in India.

## METALLIC MINERALS IN INDIA

Minerals are vital raw materials for industrial development. In India, systemic exploration is undertaken by the Geological Survey of India (GSI), Indian Bureau of Mines (IBM), State Directorate General Mining (DGM), and other undertakings of the State and Central governments. Mining and mineral-based industries have registered a significant growth over the last 50 years. Minerals found in India are classified as metallic and nonmetallic. The metallic group consists of the ferrous group that includes Iron, Manganese, Chromium; and the non-ferrous group which comprises Aluminium, Copper, Lead, Zinc, etc.[1]

### Ferrous group of metallic minerals

The ferrous group of minerals includes manganese, chromite, and iron. The contribution of iron ore in the gross domestic product is about 5%, whereas that of chromite is about 1%. Iron ore is a basic raw material that is chiefly used for the making of steel, sponge iron, and pig iron. The industrial progress and military potential of a country are judged by its per capita consumption of iron metal. Chromite is used in the manufacture of chromium metal and alloys; it imparts additional strength, hardness, and toughness to its alloys. Manganese ore is an important material for the iron and steel industry; it improves the strength and toughness of steel. Table 1 shows details of ferrous group metals, the chemical composition of the ore, geographical location of the mines and subjects exposed during the mining process.[1,2,3]

**Table 1:** Ferrous group of metallic minerals

Mineral	Ore	Chemical Compositions	Mining states	No. of Mines	No. of Workers
Chromium	Chromite	Cr_2_O_3_ (44–48%),	Orissa	19	4,387
		Fe_2_O_3_ (16–30%),	Karnataka, MS, A P,		
		Al_2_O_3_ (15%) MgO			
		(12%), Ti (t), V(t)			
Iron	Hematite	Fe_2_O_3_ (60–65%),	A P, Jharkhand,	247	34,568
	Magnetite	SiO_2_ (13%),	Karnataka, CG,		
		Al_2_O_3_ (3%),	Bihar, MP,MS,		
		P (0.1%)	Goa, Raj, UP,		
			Orrissa,		
Manganese	Pyrolusite,	MnO(25–45%),	MS, MP, Orissa,	119	12,735
	Psilmelane,	SiO_2_ (13%),	A P, Jharkhand,		
	Cryptomelane	Al_2_O_3_ (7.5%),	Raj, Karnataka		
	Braunite	P(0.2%),	Cu (t), Pb(t),		

The exact number of mines and subjects employed may vary, Chemical composition of ore represents the constituents broadly, Data of atomic mineral excluded, (t)- Trace, MS-Maharashtra, MP-Madhya Pradesh, AP-Andhra Pradesh, Raj-Rajasthan, CG-Chattisgarh, UP-Uttar Pradesh

### Nonferrous metallic minerals

Nonferrous metallic minerals chiefly include aluminium, copper, and lead. Aluminium is widely used in transport (it is replacing steel in the automobile industry), construction, food packaging, and chemical industries. India is a pioneer in replacing copper by aluminum in power transmission and distribution. Lead is among the most widely used nonferrous metals in the world. The battery sector is known for prime lead consumption. Copper has many industrial applications. Table 2 presents details of the nonferrous group metals with respect to chemical composition of the ore, exposed subjects, and geographical locations of the mines.[1,2]

**Table 2:** Nonferrous metallic minerals

Mineral	Ore	Chemical Compositions	Mining states	No. of Mines	No. of Workers
Aluminium	Bauxite	Al_2_O_3_ (55%),	Orissa, AP,	204	9,117
		Fe_2_O_3_ (4.5%), Ti (t),	CG, Gujarat,		
		P(t), V(t), F(t)	Jharkhand		
Copper	Copper sulphate	Cu S (69.5%),	M P, A P,	6	2,824
	chalcocyte chalcopyrite	Fe_2_O_3_ (30.4%), Hg (t),	Sikkim,		
		As (t), Pb(t), Cd(t)	Jharkhand		
Gold	Gold ore	Al_2_O_3_ (6.1%),	Karnataka,	3	3,773
		Fe_2_O_3_ (4.62%),	Jharkhand,		
		Au (t), Hg(t)	AP,MP, CG		
Lead	Lead - zinc ore,	Pb (17.1%),	Raj, M P,Bihar	7	2,664
	Galena, anglesite,	Zn(35.7%),			
	cerussite	Cd (1.5%), Cu(t),		
		Mn (t), As (t)			

The exact number of mines and subjects employed may vary, Chemical composition of ore represents the constituents broadly, Data of atomic mineral excluded, (t)- Trace, MS-Maharashtra, MP-Madhya Pradesh, AP-Andhra Pradesh, Raj-Rajasthan, CG-Chattisgarh, UP-Uttar Pradesh.

## HEALTH HAZARDS OF COMMON TOXICANTS IN THE MINING ENVIRONMENT

Mine workers are exposed to dust having different concentrations of toxic materials in the course of their work. Inhalation and absorption through the skin are the common routes of exposure. Whereas acute toxicity is rare in the mining industry, low-dose chronic exposure may result in insidious poisoning that is detectable only through biological monitoring in the early stages. Exposure to increasing amounts of toxic substance(s) over long periods leads to the accumulation of toxicants in the body that may manifest clinically at a very late stage.

Lead is an important toxicant in the mining environment; it is absorbed mainly by inhalation and to small extent through the gut and the skin. Early clinical features of long-term exposure are abdominal colic, obstinate constipation, loss of appetite, blue line on the gums, insomnia, headache, mental confusion, delirium etc. Chronic exposure to inorganic lead primarily affects erythropoiesis, resulting in a combination of anemia and bone marrow erythroid hyperplasia. The most common biological samples for lead monitoring are blood and urine. Hair and nail samples provide information about past exposure.[4] Blood lead level should be < 10 *µ*g/dL,[5] if greater than this level, the worker should be removed from further exposure. The OSHA (Occupational Safety and Health administration) standard also requires the measurement of zinc protoporphrin (ZPP) as part of the biological monitoring program. Measurements are to be carried out of urinary coproporphyrin, urinary δ-aminolevulinic acid (ALA), and erythrocyte ALA and its related enzymes in red blood cells. δ-aminolevulinic dehydratase (ALAD) is also a useful marker of the effects of lead.[4]

Manganese exposure in mine workers occurs mainly via inhalation of dust, absorption through the skin, and ingestion. Early clinical features are anorexia, asthenia, apathy, somnolence, headaches, etc. A few may experience a brief period of aggressiveness, increased sexual activity, and hallucinations. The toxicity may be manifested as a chronic disorder of the central nervous system (CNS) resembling Parkinson's disease. Toxicity to the lung is manifested as an increased susceptibility to bronchitis or in serious cases, may cause manganic pneumonia. Concentration > 10 *µ*g/L blood is suggestive of manganese exposure. Estimation in hair also gives an idea about past exposure.[6] The rapid clearance of manganese from the blood limits its usefulness as a biomarker of toxicity. In addition, blood manganese levels are influenced by the accumulated body burden and therefore, may not accurately reflect an immediate past exposure and recently acquired uptake. Despite these limitations, blood levels have been used for monitoring manganese exposure. As yet, no fully satisfactory biomarker of exposure to manganese has been identified. Magnetic resonance imaging (MRI) detects increased deposition of manganese in the basal ganglia of manganese-exposed individuals.[6]

Aluminium dust is absorbed through inhalation. Chronic exposure to aluminium dust causes respiratory disorders like asthma, chronic bronchitis, pulmonary fibrosis, and granulomatous lung disease. Some studies have reported high concentrations of aluminium in the brains of patients with Alzheimer's disease while others have not.[7,8] Potroom palsy observed in aluminium smelters is a progressive neurological disorder characterized by incoordination, poor memory, impairment in abstract reasoning, and depression.[9] Normal aluminium concentration in blood is < 5.4 *µ*g/L and in urine 5–30 *µ*g/L. Biological monitoring of exposure to aluminium is limited to quantification of aluminium in blood and urine. Computed tomography (CT) and Magnetic resonance imaging (MRI) can be used to demonstrate cortical atrophy and enlarged ventricles in a suspected case of Alzheimer's disease, and to differentiate the cases of metabolic, vascular, and other etiologies of dementia from those of neurotoxic origin.[10]

Fluoride is poorly absorbed from intact skin but readily absorbed from the lung and the gastrointestinal tract; it accumulates in the body at levels of 4 mg/day. Chronic exposure to fluoride leads to skeletal fluorosis, resulting in an increased bone density associated with skeletal deformities and spinal rigidity. Skeletal changes as well as musculoskeletal complaints are observed in highly exposed groups. Normal whole blood level of fluoride is < 0.5 mg/L; a fluoride level of 4 mg/L in urine is considered the upper limit of normal levels; levels above 4 mg reflect excessive fluoride exposure and those above 7 mg are considered to lead to fluorosis.[11] Finger nails and urine can be used as biomarkers for fluoride exposure.[12]

Mercury in the mining environment is absorbed mainly by the lung and to a small extent by the gut and the skin; it is deposited in the brain, kidney and liver. Clinical features of chronic exposure are weakness, fatigue, insomnia, allergic skin rash, loss of appetite, inflammation of the gums, and impaired memory. Psychological disturbances consisting of nervousness, irritability are seen besides peripheral neuropathy that normally affects the lower extremities.[13] The magnitude of exposure to mercury is usually monitored in terms of total mercury in the blood and urine; no other specific biomarkers have been suggested. Normal blood mercury levels are 0.6–59 *µ*g/L and urine levels are < 20 *µ*g/L.[14] Estimation in hair provides information about past mercury exposure.[13]

Arsenic is released during the smelting process of copper, zinc, tin, and lead. It is an irritant to the skin and may cause eczematous dermatitis and arsenical keratosis usually on the palms and soles on contact. Chronic toxic effects include megaloblastic bone marrow suppression and liver enlargement but may also affect higher nervous functions like cognition and personality changes.[15] Normal blood concentration is 2–23 *µ*g/L and urine levels are 5–50 *µ*g/d. Biological monitoring of arsenic is usually conducted by measuring total arsenic in the hair, nails, or urine. Biological monitoring of arsenic exposure by measurements in the hair and nails is complicated by potential external contamination problems, and adsorption of arsenic on to the surface of these matrices. Measurement of arsenic in blood is not routinely used for biological monitoring because urinary measurements are more reliable and easily obtained. The measurement of arsenic in urine is the most common matrix for biomonitoring because urinary elimination is the major route of excreting arsenic from the body. Monomethyl arsenic acid and dimethylarsinic acids are the major urinary metabolites.[16]

Cadmium is a byproduct of mining and smelting of lead and zinc. Cadmium is poorly absorbed from the gut and only 6% of the ingested dose is taken up by the body, whereas up to 40% is retained in the lung. Chronic low-dose cadmium exposure leads to proximal renal tubular defects which are irreversible. Normal blood cadmium level is < 10 *µ*g/L and normal urine level is < 2 *µ*g/g of urinary creatinine.[17] For the biological monitoring of cadmium exposure, cadmium concentrations in blood and urine are the most important parameters. The level of cadmium in blood can be considered as an indicator of current exposure, whereas cadmium in urine reflects the cadmium body burden in the absence of renal damage. Biological monitoring allows a direct assessment of the risk of kidney dysfunction in individual workers exposed to cadmium. Measurements of selected proteins and enzymes in urine can be used to assess the effects of cadmium on renal function. Urinary retinol binding protein and β_2_-microglobulin are presently recognized as the best markers for the early detection of tubular dysfunction due to cadmium.[18]

## MAGNITUDE OF TOXICITY RISK

Not many studies are available from India to make accurate extrapolations regarding the number of Indian miners at risk of toxicity. The number of subjects with probable toxicity among Indian miners has been determined upon the basis of reported studies of miners from India as well as abroad. Table 3 shows the estimated cases of Indian miners with apparent/subclinical toxicity vis-à-vis different toxic substances.

**Table 3:** Estimate of Indian metallic miners at risk of toxicity

Toxicant	Nature of mine	No. of exposed miners	Assumption of toxicity	Estimated cases
Lead	Copper, Lead- Zinc, Manganese	18,223	12%*[19]	2186
Mercury	Copper, Gold, Lead-zinc	9261	13%*[20]	1204
Arsenic	Copper, lead-zinc	5488	21%*[21]	1152
Cadmium	Copper, Lead- zinc	5488	10%*[17]	548
Aluminium	Bauxite, Chromium, Manganese, Gold, Iron,	64580	10%*[22]	6458
Fluoride	Aluminium	9117	13%*[23]	1185
Manganese	Manganese, Chromite, Lead	19786	20%**[24]	3957

* data from outside India

** Indian data

The clinical features of chronic exposure often manifest at a very late stage and could be nonclassical because of multiple toxicant exposure. Hence, the resultant effects of multiple toxicants need to be fully elicited. Table 3 shows the estimated number of mine workers with clinical/subclinical toxicity. It can be presumed that many of these subjects have elevated blood levels/body burden of toxicants with vague/no symptoms. The health check-ups of the workers of the mining industry are carried out periodically but they do not include biological monitoring. The large unorganized sector employs many workers but contributes little to the production of metallic minerals and in absence of reliable information regarding the number of workers engaged, they have not been considered in this article.

Dust and free silica monitoring is carried out periodically as per the norms of the Directorate General Mining Safety (DGMS), India, and among toxicants, manganese monitoring is mandatory in manganese mines.[25] However, it is essential to make provisions to monitor other toxicants as well in the mining environment and also among workers of the organized and unorganized sectors for toxic levels. Thus, there is a need to find out the exact number of mineworkers suffering from toxicity and devise suitable strategies for prevention and control in India.

## CONCLUSIONS

Mine workers are exposed to dust of various toxic materials. The major toxicants in mining environment are lead, manganese, mercury, arsenic, and cadmium but very little information about the health impact of these toxicants and its magnitude is available in current scientific/regulatory reports.

The clinical manifestations are often nonclassical because of subclinical toxicity and the possibility of multitoxicant exposure. The resultant effect needs to be fully elicited.

It is essential to monitor toxicants in the environment and mine workers for toxic levels of substances so that cases of subclinical poisoning may be identified at an early stage.

## RECOMMENDATIONS FOR IMPROVING MINERS' HEALTH SERVICES IN INDIA

- Dust monitoring of work area should include characterization and quantification of toxic substances in mine environments.
- Periodical medical examination should include estimation of such toxicants or their metabolites in appropriate body tissues.
- Awareness regarding prevention of toxicant-related health hazards in the mining industry should be created in the mines' managements by conducting training and education programs.

Specific rules should be framed for monitoring the toxicants in mine environments and exposed workers.
